# Assessment of factors influencing retention of health workforce in rural and remote areas of Odisha, India

**DOI:** 10.1186/1753-6561-6-S5-O4

**Published:** 2012-09-28

**Authors:** Shridhar Kadam, Sanghamitra Pati, Mohammad Akhtar Hussain, Srinivas Nallala, Nayan Chakravarty, Bhuputra Panda, Biswamitra Sahu, Abhimanyu Singh Chauhan, Shomik Ray, Sangram Swain

**Affiliations:** 1 Indian Institute of Public Health, Bhubaneswar, Odisha, India; 2 Indian Institute of Public Health, Delhi, India; 3 State Human Resource Management Unit, Odisha, India

## Introduction

The scarcity of qualified health workers in rural areas is directly affecting delivery of health services and their quality. Diverse interventions have been instituted by central and state governments to attract health workers to rural areas and to enhance the retention of qualified workers in India. However the reasons for not willing to remain in rural and remote areas are still poorly understood. This study explored factors influencing health workers’ retention in rural and remote areas of Odisha.

## Methods

We carried out the study in six districts of Odisha selected randomly from three geographic and administrative regions of the state. We used a mixed methods approach to collect both quantitative and qualitative data. A total of 226 semi-structured interviews were conducted with doctors, nurses, pharmacists, multipurpose health workers (MPHW) and laboratory technicians. A multi stage stratified random sampling was used for selecting study participants working at different levels of health facilities i.e. sub centres, primary health centres and community health centres.

## Results

We found that except a few districts, the ratio of female MPHW to population was around 5000 in the state, which is at par with the prescribed norms of government of India. The ratio of government allopathic doctor, laboratory technician and staff nurses to population are: 13000, 40000 and 15000, respectively.

Majority of health staffs perceived “strong personal will to serve people”, “physical infrastructure”, “training opportunities”, “support by seniors”, “good schooling for their children” and “promotion avenues after certain years of rural service” as important factors for continuing to work in rural and remote areas.

Most of the participants were found to be satisfied with the respect and trust received from their patients. The support received from the seniors in the health bureaucracy and the local community was also found to be satisfactory.

The major reasons for dissatisfaction that were attributed by study participants to work in rural areas included existing promotional avenues following the terms of rural service, lack of physical infrastructure and schooling facility for the children of the health staff. Five primary reasons ranked in order of priority cited by the study participants for continuing at the same place were, namely, permanent government service, pension facility, social service, source of regular income and job satisfaction.

## Discussion

Professional growth in the form of promotion and skill development, suitable physical infrastructure at workplace and schooling for their children along with additional monetary incentives were the key and inter-relating factors influencing the retention of health workforce in rural and remote areas. Hence a combination of interventions like monetary incentives with enhanced career opportunities for professional growth (training, higher studies and promotion), scholarships and preference of seats in reputed (residential) schools to the children of staff and suitable physical infrastructure at workplace would be more effective than financial incentives alone. There is a need for clearly defined human resource policy for health personnel across all cadres with defined parameters for performance appraisal, transfer and promotion.

## Competing interests

Authors declare that they have no conflict of interest.

## Funding statement

This study was funded by the Technical Management Support Unit, Odisha.

